# Mammary duct ectasia in adult females; risk factors for the disease, a case control study

**DOI:** 10.1016/j.amsu.2021.01.023

**Published:** 2021-01-18

**Authors:** Ayad Ahmad Mohammed

**Affiliations:** Department of Surgery, College of Medicine, University of Duhok, Nakhoshkhana Road, 8 AM-1014, Duhok, Kurdistan Region, Iraq

**Keywords:** Duct ectasia, mammary duct ectasia, Mastalgia, nipple discharge, benign breast diseases

## Abstract

**Introduction:**

Mammary duct ectasia is a common clinical condition characterized by abnormal dilatation of the central milk ducts with chronic inflammation and fibrosis, it may affect one or both breasts. Patients may be completely asymptomatic or have mastalgia or nipple discharge which is usually from multiple ducts. It mostly affects females and is very rare in males.

**Patients and methods:**

This is a case control study which included 236 females grouped into two equal groups, the first group were patients with duct ectasia compared and the other one apparently healthy females and both groups were compared regarding different characteristics.

**Results:**

Most patients were young with a mean age of 35 years, a most of them were overweight (42.4%) and obese (33.1%). Most were menstruating (86.4%) with regular cycles (79.7%). Most patients had breast pain (67.8%), tenderness (54.2%), and no nodularity (98.3%). About 47.5% had nipple discharge mostly from multiple ducts (43.2%), 52.5% had no discharge. There was a significant correlation between the development of duct ectasia and each of marital status, lactational status, coffee consumption, pain, nodularity, and breast tenderness (P values 0.026, 0.016, 0.034, 0.000, 0.000, and 0.000).

**Conclusion:**

Duct ectasia is a very common complaint in females, it is commoner in overweight and obese females, married females and those with history of lactation. Coffee consumption may be a cause. The regularity of the menstruation has no correlation with its development. The presence of mastalgia, tenderness, and nodularity are highly suggestive for the disease.

## Highlights

• The breast is a dynamic structure that undergo various stages of development.
• Mammary duct ectasia is a common clinical condition.
• It may affect single breast or both breasts.
• It is common in obese females, married females and those with history of lactation.
• Patients have mastalgia, breast tenderness, and nodularity.

## 1 Introduction

The breast is a dynamic structure that undergo various stages of development during the female's reproductive life such as puberty, pregnancies, lactation, and menopause, these collectively results in many changes in the architecture of the breast structure [1].

Mammary duct ectasia is defined as abnormal dilatation of the central milk ducts associated with chronic inflammation and fibrosis. It is a common clinical condition, and it may affect single breast or both breasts [[2], [3], [4]].

The condition was first described in 1951, the etiology is still not very clear, the debate is still present regarding whether periductal mastitis is the cause or the result of mammary ductal dilatation. The condition is mostly bilateral and exhibit a chronic relapsing and remitting course [3,5].

Patients may be completely asymptomatic or may present with mastalgia and or nipple discharge, the discharge is usually from multiple ducts, although some patients have discharge from multiple ducts. The color of the discharge is variable, it may be greenish in color, yellowish in color or sometimes blood stained, bloody nipple discharge may be associated with breast cancer or intra-ductal papilloma and such possibilities must be excluded. It mostly affects females, the disease is very rare in males [3,[6], [7], [8]].

Chronic cases develop chronic subareolar abscesses and chronic fibrosis which result in increasing pain, palpable tender masses, fever, nipple retraction, and fistula formations. In chronic cases the consistency of the discharge change from a thin discharge to more thick and cheesy texture due to cholesterol crystal precipitation [3].

The differential diagnoses of mammary duct ectasia include benign breast cysts, fibrocystic disease, and malignancy especially when there is mass and nipple retraction. The differentiation is usually done with the aid of imaging and sometimes by histopathology. Benign breast cysts are usually seen in terminal parts of duct lobular units, in contrast to duct ectasia in which the subareolar ducts are involved but this is not critical in distinction. Juvenile papillomatosis is a rare localized lesion which involves ductal stasis, intraluminal histiocytes, florid duct hyperplasia, papillary proliferations, and sclerosis. Ductal hyperplasia and papillary epithelial hyperplasia are not present in mammary duct ectasia [4,9,10].

The evaluation of the patients is usually done adopting the triple assessment, it include an appropriate history taking and clinical examination, the use of imaging modalities depending on the clinical findings, ultrasound and mammography are the most widely used imaging techniques for the evaluation of breast lesions. The sonographic features depend on the stage of the disease but usually shows duct dilatation, while mammography shows features of benign breast calcifications like branching calcifications and retroareolar duct dilatation. Some cases require more complex imaging techniques like CT-scan or MRI. Ductoscopy is also helpful in the diagnosis especially when the discharge is from a single duct [[4], [5], [6],^11^].

Other part of the triple assessment is the tissue examination or sometimes the cytology. Nipple discharge cytology may be required in some cases but usually more details of the lesions may be required which make biopsy more appropriate [6].

The tissue examination showed various degrees of inflammation, infiltration with chronic inflammatory cells particularly plasma cells, and foam cell formation, breast duct dilatation, periductal fibrosis, intraepithelial histiocytes, calcifications in duct lumen or duct wall, and abscess formation [3,4,12].

The management depend on the stage of the disease and the nature of the presentation, when there is infective complications patients may require antibiotics and drainage of abscesses, severe cases may require excision of the affected ducts is usually required, ductoscopy if available will help to diagnose the only affected ducts and avoid unnecessary duct excision. Some refractory cases may require some forms of reconstructive surgery. Recently, some forms of new modalities of treatment are tried which may help to stop the progression of the disease, these agents act by reducing the release of inflammatory pathways mediated by IL-6 and reducing the expression of Bcl-2 [[13], [14], [15], [16], [17]].

## 2 Patients and methods

This is a case control study which included 236 individuals who were grouped into two groups, the first group included 118 female patients who were presented to the breast clinic consecutively and diagnosed with duct ectasia compared to 118 apparently healthy females. Both groups were compared regarding different characteristics.

Patients who were diagnosed with other benign breast pathologies other than duct ectasia, those with breast cancer, patients with insufficient data, and male patients were excluded.

The correlations were calculated using the two sided asymptomatic significance, P values equal or below 0.05 were considered significant. For categorical data, the Pearson Chi square test were used and the independent *t*-test were used for the numerical ones. Data were analyzed using the statistical Package for Social Sciences (SPSS, 25).

The manuscript has been registered at the research registration unit of the Duhok Medical College and approval is granted with a registration number: 3 N at 6-12-2020.

In accordance to the World Medical Association's Declaration of Helsinki 2013, the work of this article is registered in the Research Registry, and the unique identifying number is: researchregistry 6335.

The link to the registration page is: https://www.researchregistry.com/browse-the-registry#home/registrationdetails/5fcb39101872aa001b2df9ee/

The work of this article has been reported in line with the STROCSS criteria [18].

## 3 Results

Most patients involved in our study were young patients with a mean age of 35 years, and most of them were overweight and obese. Most were menstruating with regular cycles. Other general characteristics are shown in Table 1.

**Table 1:** The charecteristics of the patients.

Category	Subcategories	Frequency	Percentage
Age (M;SD) Range: 20-50		35.81	8.760
BMI (M;SD) Range: 20-41		28.36	4.276
BMI values	Healthy weight (18.5–24.9)	22	18.6
	Over weight (25–29.9)	50	42.4
	Obese (30–34.9)	39	33.1
	Severely obese (35–39.9)	5	4.2
	Morbidly obese (≥40)	2	1.7
Menarche (M;SD) Range: 10–16 years		12.53	1.068
Menstrual status	Menstruating	102	86.4
	Menopausal	16	13.6
Menstrual cycles	Regular	94	79.7
	Irregular	24	20.3
Marital status	Married	112	94.9
	Single	6	5.1
Age at 1st pregnancy (M;SD) Range: 13–30 years		20.79	3.276
Number of children (M:SD) Range: 0–12 children		3.15	2.225
Lactation status	Lactating	8	6.8
	Non-lactating	14	11.9
	History of lactation	96	81.4
Smoking status	Smoker	2	1.7
	Non-smoker	116	98.3
Alcohol consumption	Yes	2	1.7
	No	116	98.3
Coffee consumption	Yes	2	1.7
	No	116	98.3
OCCP	Yes	2	1.7
	No	116	98.3

Patients were assessed by the clinical history taking breast examination, to assess for any clinical signs particularly tenderness and nodularity. The nipples were also assessed for any nipple discharge and when there was detected, we assessed whether the discharge is from single or multiple ducts. Most patients had breast pain, tenderness, and no nodularity. About 47.5% had nipple discharge, mostly from multiple ducts. Table 2.

**Table 2:** The symptoms and signs detected during examination of the patients.

Category	Subcategories	Frequency	Percentage
Pain	Yes	80	67.8
	No	38	32.2
Nodularity	Yes	2	1.7
	No	116	98.3
Breast tenderness	Yes	64	54.2
	No	54	45.8
Nipple discharge	Yes	56	47.5
	No	62	52.5
Ducts	Single duct	5	4.2
	Multiple ducts	51	43.2
	No discharge	62	52.5

**Fig. 1:**
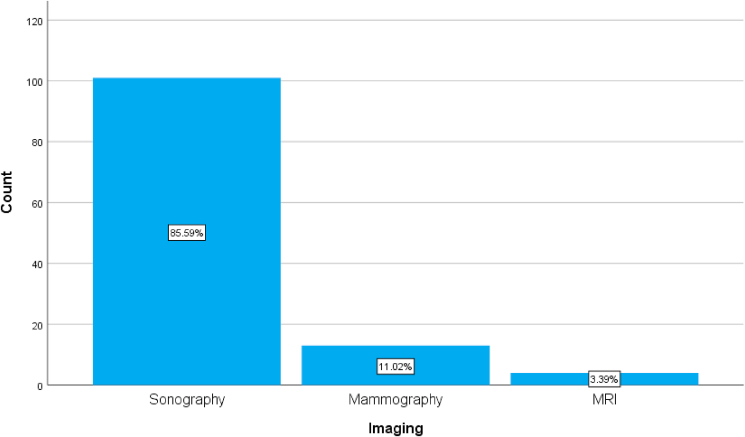
A simple bar chart showing the different imaging modalities useed to diagnose duct ectasia.

Patients were assessed some of the imaging techniques like breast ultrasound, mammography, and some patients were assessed by magnetic resonance imaging. Fig. 1.

The correlations were assessed by comparing normal patients with those who were diagnosed with duct ectasia, for the categorical data we used the Fischer Exact test and the Pearson Chi square test. There was a significant correlation between the development of duct ectasia and each of marital status, lactational status, coffee consumption, pain, nodularity, and breast tenderness. Table 3.

**Table 3:** Showing the correlation for categorial data between duct ectasia and different charecteristics, symptoms, and signs.

Category	Subcategories	Duct ectasia		Sig. (2-sided)
		No duct ectasia (controls) (n = 118)	Yes (n = 118)	
BMI values	Healthy weight (18.5–24.9)	24(20.3%)	22(18.6%)	0.831^a^
	Over weight (25–29.9)	52(44.1%)	50(42.4%)	
	Obese (30–34.9)	32(27.1%)	39(33.1%)	
	Severely obese (35–39.9)	8(6.8%)	5(4.2%)	
	Morbidly obese (≥40)	2(1.7%)	2(1.7%)	
Menstrual status	Menstruating	97(82.2%)	102(86.4%)	0.474^b^
	Menopausal	21(17.8%)	16(13.6%)	
Menstrual cycles	Regular	93(78.8%)	92(78.0%)	1.00^a^
	Irregular	25(21.2%)	26(22.0%)	
Marital status	Married	101(85.6%)	112(94.9%)	**0.026**^b^
	Single	17(14.4%)	6(5.1%)	
Lactation status	Lactating	2(1.7%)	8(6.8%)	**0.016**^b^
	Non-lactating	27(22.9%)	14(11.9%)	
	History of lactation	89(75.4%)	96(81.4%	
Smoking status	Smoker	9(7.6%)	2(1.7%)	0.059^b^
	Non-smoker	109(92.4%)	116(98.3%)	
Alcohol consumption	Yes	1(0.8%)	2(1.7%)	1.00^a^
	No	117(99.2%)	116(98.3%)	
Coffee consumption	Yes	10(8.5%)	2(1.7%)	**0.034**^b^
	No	108(91.5%)	116(98.3%)	
OCCP intake	Yes	5(4.2%)	2(1.7%)	0.446^a^
	No	113(95.8%)	116(98.3%)	
Nipple discharge	Yes	4(3.4%)	56(47.5%)	**0.000**^b^
	No	114(96.6%)	62(52.5%)	
Pain (mastalgia)	Yes	109(92.4%)	78(67.2%)	**0.000**^a^
	No	9(7.6%)	38(32.8%)	
Nodularity	Yes	24(20.3%)	2(1.7%)	**0.000**^a^
	No	94(79.7%)	114(98.3%)	
Breast tenderness	Yes	101(86.3%)	62(53.4%)	**0.000**^a^
	No	16(13.7%)	54(46.6%)	

a Fischer Exact test.

b Pearson Chi square test.

For the numerical data we used the independent *t*-test to detect any correlation, there was no positive correlation with the age, age at the first pregnancy, and the number of children. Table 4.

**Table 4:** Showing the correlation for numerical data between duct ectasia and different charecteristics, symptoms, and signs.

Category	Mean Difference	Std. Error Difference	95% Confidence Interval of the Difference		Sig. (2-tailed)
			Lower	Upper	
Age	1.373	1.389	−1.363	4.109	0.324
Menarche	0.042	0.125	−0.204	0.289	0.735
Age at 1st pregnancy	0.191	0.487	−0.770	1.151	0.695
Number of children	0.237	0.328	−0.408	0.883	0.470

## 4 Discussion

Mammary duct ectasia is one of the commonest causes of benign breast disorders which affects females, it is usually underestimated by most physicians, and there is no very clear guidelines for its management. There still a great debate regarding whether plasma cell mastitis and mammary duct ectasia are similar or two clinical and pathological entities [8,13].

Most benign breast disorders are more common at young ages particularly in the second and the third decades of life, in the current study the mean age of the patient were 35.81 years, and 86.4 were menstruating, most of them (79.9%) of them had regular cycles. Late menarche may have a possible correlation with the development of future mammary duct ectasia, in the current study, this like was not present (P value 0.735) [19,20].

The diagnosis of mammary duct ectasia is merely sonographic and there is usually there is no diagnostic difficulty with the experienced hand, rarely some cases may require further evaluation by mammography or even MRI, in our study about 85.59% of cases were diagnosed with ultrasound, some cases required mammography and MRI (11.02% and 3.39%) respectively [3].

Some studies implicate age as one of the risk factors for the development of many benign breast disorders, the age was not a risk factor in our study, and the correlation was not significant when compared to the controls (P value 0.324). Obesity also have been implicated by some authors to have a possible association, although in this study this correlation was not present (P value 0.831) [6,12].

In our study the parity of the patient had no positive correlation with the development of mammary duct ectasia (P value 0.470), similar results were proved in some other similar articles [2].

Most cases with blood negative nipple discharge had benign breast disorder, the most common of them is duct ectasia, and in our study 47.5% of our patients had nipple discharge mostly from multiple ducts, the correlation was very significant with duct ectasia (P value 0.000). Cases of bloody nipple discharge must be further evaluated to exclude a more serious cause, and in cases when there is suspicion of blood in the discharge like black or brown discharge, it should be sent for evaluation for the presence of red blood cells [6].

Smoking is claimed to have a strong association with the development of duct ectasia by some studies, however in the current study the association with smoking was not significant (P value 0.059). Similarly, alcohol consumption had no correlation with the development of duct ectasia (P value 1.00). Coffee consumption is a matter of great investigations whether or not it is associated with the development of benign breast disorders including duct ectasia, in our study, duct ectasia has a significant correlation with increasing coffee consumption (P value 0.034), however most studies correlated this to the development of mastalgia rather than duct ectasia [[20], [21], [22]].

During examination most patients have pain and tenderness, nodularity may be felt during examination, and these three findings are strongly associated with the diagnosis of duct ectasia and may be strongly suggestive for the disease (P values 0.000) for each of them. A subareolar mass and tenderness is a frequent finding, abscess must be excluded and the appropriate management given [20].

Marital status is shown to have strong association in our study (P value 0.026), similarly lactation had a strong association (P value 0.016), and age of the first birth have no relation to the development of duct ectasia in our study (P value 0.695). Pregnancy and lactation are associated with increase the size of the milk ducts and increase the risk for the development of stasis and infection [12].

The intake of oral contraceptive pills doesn't increase the risk for the development of duct ectasia among our patients (P value 0.446), this fact is proved y some other authors supporting our findings. The intake of oral contraceptive pills have been linked to the development of mastalgia and fibrocystic disease [11].

Some limitations of the study include a relatively small sample size, and some patients were excluded because of limited access to information.

## 5 Conclusion

Duct ectasia is a very common complaint in females, it is commoner in overweight and obese females, married females and those with history of lactation. Coffee consumption may be a cause. The regularity of the menstruation has no correlation with its development. The presence of mastalgia, tenderness, and nodularity are highly suggestive for the disease. Reassurance of patients is a very important part in the management and long follow up is not usually recommended.

## Ethical approval

NA.

## Sources of funding

No source of funding other than the authors.

## Author contribution

Study design, data collection and analysis, writing and final approval of the manuscript: Dr Ayad Ahmad Mohammed.

## Trial registry number

N/A.

## Research registration unique identifying number (UIN)

Researchregistry6335.

## Guarantor

Dr Ayad Ahmad Mohammed.

## Provenance and peer review

No commissioned, externally peer-reviewed.

## Declaration of competing interest

No conflicts of interest present.
